# Targeting *RASL12* by miR-6791-5p fuels malignant progression in lung adenocarcinoma

**DOI:** 10.1186/s41065-026-00697-4

**Published:** 2026-07-25

**Authors:** Lu Zhang, Zhonghao Pang, Haibo Zhang, Pin Wang

**Affiliations:** 1 Department of Infectious Diseases, Longyou County People’s Hospital, Quzhou City, Zhejiang Province 324400 China; 2 Department of Cardiothoracic Surgery, Affiliated Hospital of Jiangsu University, Zhenjiang City, Jiangsu Province 212001 China; 3 Department of Emergency, The Fourth Hospital of Tongxiang, Tongxiang City, Zhejiang Province 314502 China; 4 Department of Respiratory and Critical Care Medicine, Tiantai People’s Hospital of Zhejiang Province (Tiantai Branch of Zhejiang Provincial People’s Hospital), Hangzhou Medical College, No.1, Kangning Middle Road, Shifeng Street, Tiantai County, Taizhou City, Zhejiang Province 317200 China

**Keywords:** Lung adenocarcinoma, miR-6791-5p, *RASL12*, Prognosis, Metastasis

## Abstract

**Background:**

Lung adenocarcinoma (LUAD) poses significant clinical challenges due to its heterogeneity and metastatic potential, necessitating deeper molecular characterization. This study investigated the role of miR-6791-5p and its target *RASL12* in LUAD progression.

**Methods:**

Expression profiles and prognostic relevance were analyzed in 203 LUAD patients, cell lines, and public databases. Biological functions were evaluated through CCK-8, Transwell, independent *RASL12* knockdown, and rescue assays. Interactions were validated by dual-luciferase assays. Downstream pathways were explored via Gene Set Enrichment Analysis (GSEA).

**Results:**

miR-6791-5p was significantly upregulated in LUAD (*p* < 0.001), independently predicting poor overall survival (HR = 2.411, *p* = 0.003). Functionally, miR-6791-5p promoted LUAD cell proliferation, migration, and invasion. *RASL12* was confirmed as a direct target, exhibiting decreased mRNA and protein expression in tumors that negatively correlated with miR-6791-5p. Independent *RASL12* knockdown enhanced tumor aggressiveness, while its co-knockdown partially reversed the tumor-suppressive effects of miR-6791-5p inhibition. GSEA linked this axis to cell adhesion signaling dynamics.

**Conclusions:**

miR-6791-5p acts as an oncogene in LUAD by directly targeting the tumor suppressor *RASL12*. This axis modulates malignant phenotypes, representing a promising prognostic biomarker and potential therapeutic candidate.

**Supplementary Information:**

The online version contains supplementary material available at 10.1186/s41065-026-00697-4.

## Background

Globally, lung cancer is a leading cause of cancer-related morbidity and mortality [1, 2]; its most common pathological subtype, lung adenocarcinoma (LUAD), accounts for approximately 40% of all cases [3]. The long-term survival rate of LUAD patients is still not ideal due to its characteristics of concealed early symptoms and susceptibility to invasion and metastasis [4–6]. Therefore, it is of great value to explore the molecular mechanism of LUAD and to identify key molecules that can drive its malignant progression in order to develop new diagnostic markers and therapeutic targets.

MicroRNAs (miRNAs) are endogenous non-coding RNAs that function as key post-transcriptional regulators of gene expression [7, 8]; by modulating target genes, they influence essential cellular processes such as proliferation, migration, and invasion, thereby playing a pivotal role in tumorigenesis and cancer progression [9]. For example, in LUAD, miR-21-5p overexpression promotes tumor cell proliferation as well as migration and invasion [10, 11]; tumor progression is promoted by miR-941 through its targeting of *FOXN4*, which facilitates increased tumor cell migration and invasion [12]; miR-383-5p regulates tumor cell proliferation and migration by targeting the *SHMT2* gene [13]. A previous bioinformatics study by Li et al. [14] employed ceRNA network analysis to predict that the lncRNA ENST00000609697 might act as a molecular sponge for miR-6791-5p, thereby potentially regulating *RASL12* expression in LUAD. Based on this in silico prediction, the authors hypothesized that the ENST00000609697–miR-6791-5p–*RASL12* axis might play a tumor suppressor role. However, this prediction lacks experimental validation, and the specific biological function of miR-6791-5p in LUAD, as well as its direct regulatory relationship with *RASL12*, remains to be experimentally elucidated. Therefore, the present study aims to provide functional validation and mechanistic characterization of the previously predicted miR-6791-5p/*RASL12* axis in LUAD through comprehensive clinical sample analysis and in vitro functional assays.

In the complex molecular network of LUAD, abnormal activation of the RAS signaling pathway is considered to be one of the key oncogenic events. As a member of the RAS family, *RASL12* may play a unique role in maintaining cellular homeostasis. Preliminary studies have shown that *RASL12* is expressed at low levels in some tumor tissues, and its expression level is positively correlated with patient prognosis. Studies have shown that *RASL12* plays a role in bladder cancer and liver cancer [15, 16], and these findings suggest that *RASL12* may have tumor suppressor functions. However, the expression pattern, clinical significance and specific mechanism of *RASL12* in LUAD remain to be systematically elucidated.

Based on the above research background, we propose the following scientific hypothesis: miR-6791-5p may affect the malignant phenotype of LUAD cells by targeting the expression of *RASL12*, thus playing an important role in the progression of LUAD. This study will systematically explore the biological function of the miR-6791-5p/*RASL12* regulatory axis in the occurrence and development of LUAD through clinical sample analysis and a series of in vitro experiments, in order to deepen the understanding of the molecular mechanism of LUAD and provide a new theoretical basis for its precise diagnosis and treatment.

## Methods

### Study subjects

This study included a total of 203 LUAD patients from THE FOURTH HOSPITAL OF TONGXIANG. The inclusion criteria were as follows: (1) definitive diagnosis of LUAD by histopathological examination; (2) complete resection of lung cancer lesions through surgical procedures; (3) no prior anti-tumor therapy, such as chemotherapy, radiotherapy, or targeted therapy; (4) female patients were not pregnant or lactating; (5) no history of other active malignancies or autoimmune diseases within the past 5 years; (6) complete and accessible clinical data.

All included patients underwent a 60-month follow-up with assessments conducted every 3 months. Overall survival (OS) was defined as the time from the date of surgical resection to the date of death from any cause. Notably, no patients were lost to follow-up during the study period (*n* = 203). Patients who remained alive at the final follow-up endpoint were treated as right-censored observations. The Ethics Committee of THE FOURTH HOSPITAL OF TONGXIANG approved this study. All patients provided written informed consent.

### Cell culture and transfection

The human bronchial epithelial cell line BEAS-2B and LUAD cell lines (A-549, NCI-H2009, PC9, HCC827) were obtained from the Shanghai Cell Bank of the Chinese Academy of Sciences. Cells were grown in DMEM medium (Gibco, USA) containing 10% fetal bovine serum (FBS; Gibco, USA) and 1% penicillin/streptomycin (Gibco, USA) at 37 °C in a 5% CO₂ humidified atmosphere.

The hsa-miR-6791-5p mimics, specific inhibitors, and their respective negative controls (mimic-NC and inhibitor-NC) were chemically synthesized and purchased from MedChemExpress (MCE, USA; Cat. No. HY-R02147 and HY-RI02147). For *RASL12* knockdown, a specific small interfering RNA targeting human *RASL12* (si-*RASL12*), consisting of a proprietary pool of three target-specific 19–25 nt siRNAs (Cat. No. sc-90203), along with a scrambled negative control (si-NC), were purchased from Santa Cruz Biotechnology (Dallas, TX, USA). Following the seeding of logarithmic-phase cells 24 h in advance to reach 60–70% confluence, transfections were carried out using the siRNA/miRNA Transfection Reagent (MCE, Cat. No. HY-K2017) or Lipofectamine 3000™ reagent (Thermo Fisher Scientific, USA) as per the manufacturer’s protocol. The final working concentrations were 50 nM for the mimics and siRNAs, and 100 nM for the inhibitors. Transfection efficiency was subsequently validated by qRT-PCR. Subsequent to a 48 h incubation, the medium was replaced to initiate further experiments.

### qRT-PCR

Total RNA was extracted from cultured cells and tissue samples using TRIzol™ reagent (Thermo Fisher Scientific, USA) according to the manufacturer’s instructions. To quantify mRNA and miRNA expression, distinct reverse transcription strategies were employed. For *RASL12* mRNA detection, total RNA was reverse-transcribed into cDNA using the PrimeScript™ RT Master Mix (Takara, Japan) containing Oligo(dT) and random primers. For miR-6791-5p detection, cDNA was synthesized using a poly(A) tailing-based miRNA reverse transcription kit (Mir-X™ miRNA First-Strand Synthesis Kit, Takara, Japan).

Quantitative real-time PCR was subsequently performed using SYBR^®^ Premix Ex Taq™ (Takara, Japan) on a real-time PCR detection system (Applied Biosystems 7500). The PCR cycling conditions consisted of an initial denaturation at 95 °C for 30 s, followed by 40 cycles of denaturation at 95 °C for 5 s and annealing/extension at 60 °C for 30 s. A melting curve analysis was conducted after amplification to confirm the specificity of the PCR products. All reactions were performed in triplicate. The relative expression levels of *RASL12* and miR-6791-5p were normalized to the endogenous controls GAPDH and U6 snRNA, respectively, and calculated using the 2^−ΔΔCt^ method. The complete primer and oligonucleotide sequences used in this study are listed in Supplementary Table 1.

### CCK-8 assay

Cells in the logarithmic growth phase were seeded into 96-well plates at a density of 2 × 10^3^ cells per well, with 100 µL of complete medium added to each well. Measurements were taken at 0 h, 24 h, 48 h, and 72 h post-transfection. CCK-8 detection reagent (Dojindo Laboratories, Japan) was added to the wells, and following a 2 h dark incubation, the optical density (OD) at 450 nm was determined for each time point with a microplate reader (Bio-Rad xMark, USA).

### Transwell assay

Cell migration and invasion assays were performed as distinct procedures. Cells in the log phase were digested with 0.25% trypsin (Thermo Fisher Scientific, USA), resuspended in a serum-free medium (Gibco, USA), and seeded into the upper chambers of Transwell inserts (8-µm pore size; Corning, USA) at a density of 5 × 10^4^ cells per well. For the invasion assay, the upper chambers were pre-coated with 50 µL of Matrigel (1 mg/mL; BD Biosciences, USA) according to the manufacturer’s instructions. For the migration assay, uncoated inserts were used.

Before a 24 h incubation, 500 µL of complete medium (Thermo Fisher Scientific, USA) supplemented with 15% FBS as a chemoattractant was added to the lower chamber. Following the removal of non-migrated cells from the upper surface, those on the lower surface of the membrane were subjected to fixation in 4% paraformaldehyde, staining with 0.1% crystal violet (Thermo Fisher Scientific, USA), and PBS (Thermo Fisher Scientific, USA) washing. Cells were counted and imaged using an inverted microscope (Olympus IX73, Japan). Five randomly selected fields were enumerated for each sample.

### Luciferase reporter assay

The potential interaction between miR-6791-5p and *RASL12* was first bioinformatically predicted using the Target Scan database. Accordingly, the wild-type (WT) and mutant (MUT) sequences of the *RASL12* 3′-UTR, synthesized by Sangon Biotech (China), were cloned into the pmirGLO luciferase reporter vector (Promega, USA). Cells cultured in 24-well plates were co-transfected with 100 ng of the reporter constructs and either inhibitor-NC, miR-6791-5p inhibitor, mimic-NC, or miR-6791-5p mimic (at final concentrations of 100 nM and 50 nM, respectively) using Lipofectamine 3000 (Invitrogen, USA). After 48 h, luciferase activity was measured following the protocol of the Dual‑Luciferase^®^ Reporter Assay System (Promega, USA).

### Bioinformatics analysis

To comprehensively evaluate the clinical relevance and underlying mechanisms of *RASL12* in LUAD, multiple bioinformatics platforms were utilized. First, protein expression in primary LUAD and normal lung tissues was validated using the Clinical Proteomic Tumor Analysis Consortium (CPTAC) dataset via the UALCAN portal (http://ualcan.path.uab.edu/). Next, for prognostic assessment, the Kaplan-Meier Plotter database (http://kmplot.com/analysis/) was employed to analyze overall survival (OS) in an independent external cohort, categorizing patients into high and low expression groups based on the auto-selected best cutoff value. Furthermore, to identify *RASL12*-associated signaling pathways, Gene Set Enrichment Analysis (GSEA) was performed on the TCGA-LUAD dataset (*n* = 515) using the LinkInterpreter module of LinkedOmics (http://www.linkedomics.org/). The KEGG pathway database was used, with genes ranked by their Pearson correlation coefficient with *RASL12* expression. Statistical significance was defined as a nominal *p*-value < 0.05 and a False Discovery Rate (FDR) < 0.05 after 500 permutations.

### Statistical analysis

This study employed SPSS 26.0 and GraphPad Prism 9.0 for statistical analysis and graphical generation, respectively. All in vitro experiments were performed in at least three independent biological replicates (*n* = 3), with each biological replicate consisting of three technical replicates. Data are presented as mean ± standard deviation. Inter‑group comparisons were conducted using one‑way ANOVA, two‑way ANOVA, or independent‑samples t‑test, as appropriate based on the experimental design. Associations between variables were assessed by Pearson correlation analysis. To evaluate the prognostic significance of *RASL12* expression, Kaplan–Meier survival curves were generated and compared via the log‑rank test. For this purpose, a multivariate Cox regression model was also constructed to identify independent prognostic factors. Clinical variables, including tumor size, N stage, TNM stage, and differentiation grade, were selected for the multivariate Cox regression model based on their established prognostic relevance in LUAD. To ensure model stability, we assessed potential multicollinearity among these parameters using the Variance Inflation Factor (VIF). All VIF values were found to be below 2.0, confirming the absence of significant multicollinearity. Additionally, patients were classified into high- and low-expression groups for prognostic analysis based on the mean expression level of miR-6791-5p. A *p* < 0.05 was set as the threshold for statistical significance in all tests.

## Results

### The association between miR-6791-5p and demographic/clinical characteristics in patients with LUAD

A total of 203 patients with LUAD were included in this study. Based on the mean expression value of miR‑6791‑5p as the cutoff, they were divided into a low‑expression group (*n* = 94) and a high‑expression group (*n* = 109). No significant differences were observed between the two groups in age, gender, smoking history, drinking history, or lesion location (Table 1, *p* > 0.05). The distribution of patients showed significant variations with respect to tumor size, lymph node stage (N stage), TNM stage, and differentiation grade (*p* < 0.05). Specifically, the proportion of high miR‑6791‑5p expression was greater among patients with tumor size ≥ 3 cm (*p* = 0.020); among patients with lymph node metastasis (N1–N3) (*p* = 0.023); among patients with TNM stage Ⅲ (*p* = 0.018); and among poorly differentiated or undifferentiated patients (*p* = 0.036).

**Table 1:** The correlation between the expression of miR-6791-5p and clinical characteristics

Parameters	Patients (*n* = 203)	miR-6791-5p expression		*p*-value
		Low (*n* = 94)	High (*n* = 109)	
Age (years)				0.707
<60	90	43	47	
≥ 60	113	51	62	
Gender				0.462
Male	111	54	57	
Female	92	40	52	
Smoking History				0.362
no	89	38	51	
yes	114	56	58	
Drinking History				0.451
no	85	42	43	
yes	118	52	66	
Location				0.193
Left Lung	98	50	48	
Right Lung	105	44	61	
Tumor size (cm)				0.020
<3	101	55	46	
≥ 3	102	39	63	
N Category				0.023
N0	97	53	44	
N1-N3	106	41	65	
TNM stage				0.018
I-II	134	70	64	
III	69	24	45	
Differentiation				0.036
Poorly/Undifferentiated	96	37	59	
Well/Moderately differentiated	107	57	50	

N Category: Lymph Node Category; M Category: Distant Metastasis Category

*TNM stage* Tumor, Node, Metastasis Staging System

*p*-value < 0.05 indicates a significant difference

**Fig. 1:**
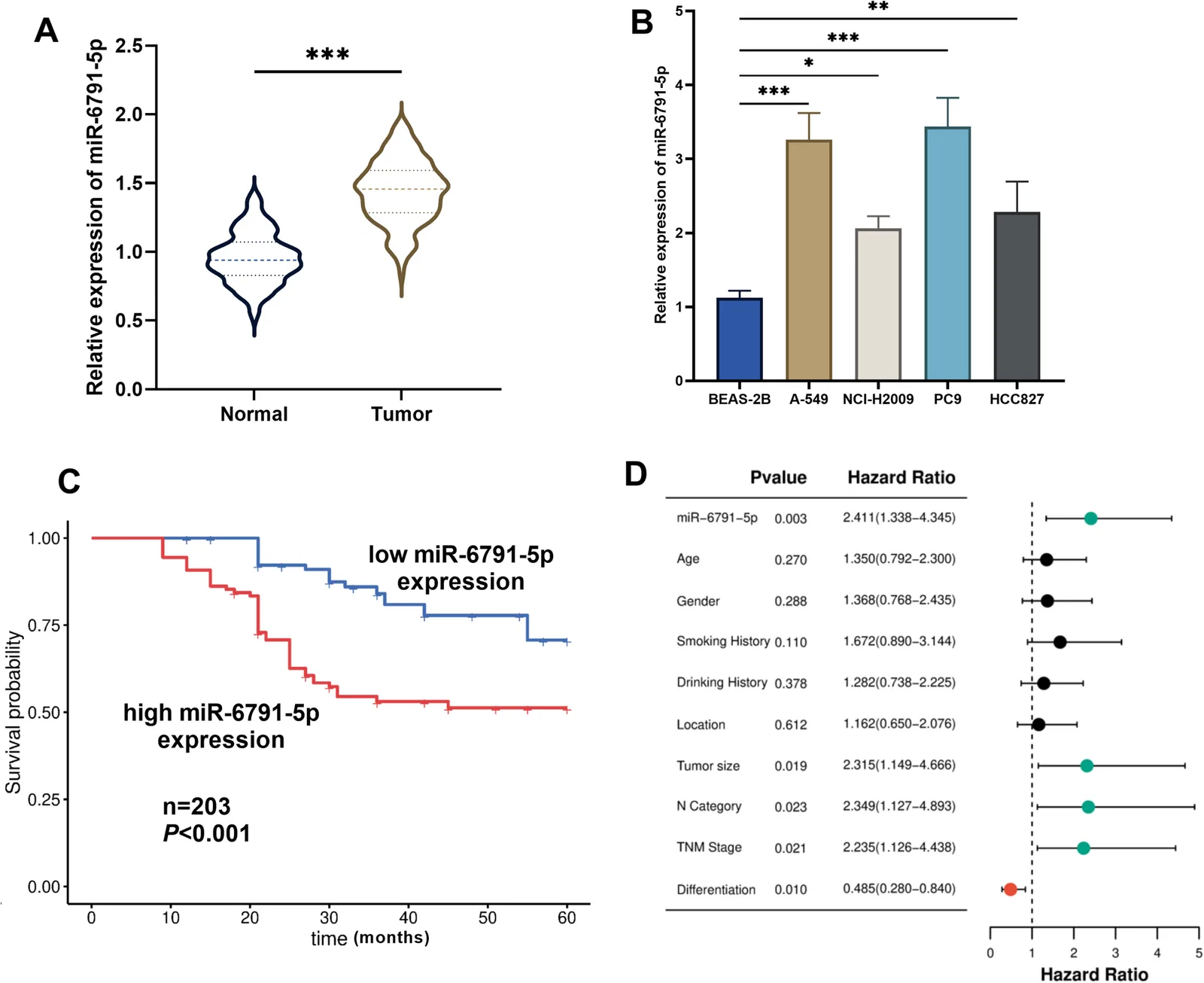
Clinical significance of miR-6791-5p in LUAD. miR-6791-5p expression in tumor tissues compared with adjacent normal tissues (**A**). Expression levels of miR-6791-5p across different cell lines (**B**). Kaplan–Meier survival analysis comparing patients with high and low miR-6791-5p expression (**C**). Forest plot of hazard ratios for prognostic factors (**D**). Data are presented as mean ± SD. Statistical significance was analyzed using paired Student’s t-test for (A) (*n* = 203), one-way ANOVA for (B) (*n* = 3 independent biological replicates), log-rank test for (C) (*n* = 203), and multivariate Cox regression for (D). *p < 0.05, **p < 0.01, ***p < 0.001

Expression analysis of 203 paired tissue samples demonstrated that miR‑6791‑5p was markedly upregulated in LUAD tissues compared to matched adjacent normal tissues (Fig. 1A, *p* < 0.001). Consistently, its expression was also elevated in multiple LUAD cell lines (A‑549, NCI‑H2009, PC9, HCC827) relative to the BEAS‑2B normal bronchial epithelial cell line, as determined by qRT-PCR analysis from three independent experiments (Fig. 1B, *p* < 0.05). Kaplan–Meier analysis of the 203 patients further indicated that high miR‑6791‑5p expression was associated with a significantly worse overall survival (Fig. 1C, *p* = 0.001).

Multivariate Cox regression identified high miR‑6791‑5p expression as an independent predictor of poor overall survival in LUAD patients (HR = 2.411, 95% CI: 1.338–4.345, *p* = 0.003). Furthermore, tumor size, N stage, TNM stage, and differentiation degree were also identified as independent prognostic factors (*p* < 0.05), whereas age, gender, and other factors had no independent prognostic value (Fig. 1D). Taken together, these clinical findings indicate that miR-6791-5p is upregulated in LUAD and serves as a robust, independent indicator of poor patient prognosis.

### The Role of miR-6791-5p in regulating proliferation, migration, and invasion of LUAD cells

**Fig. 2:**
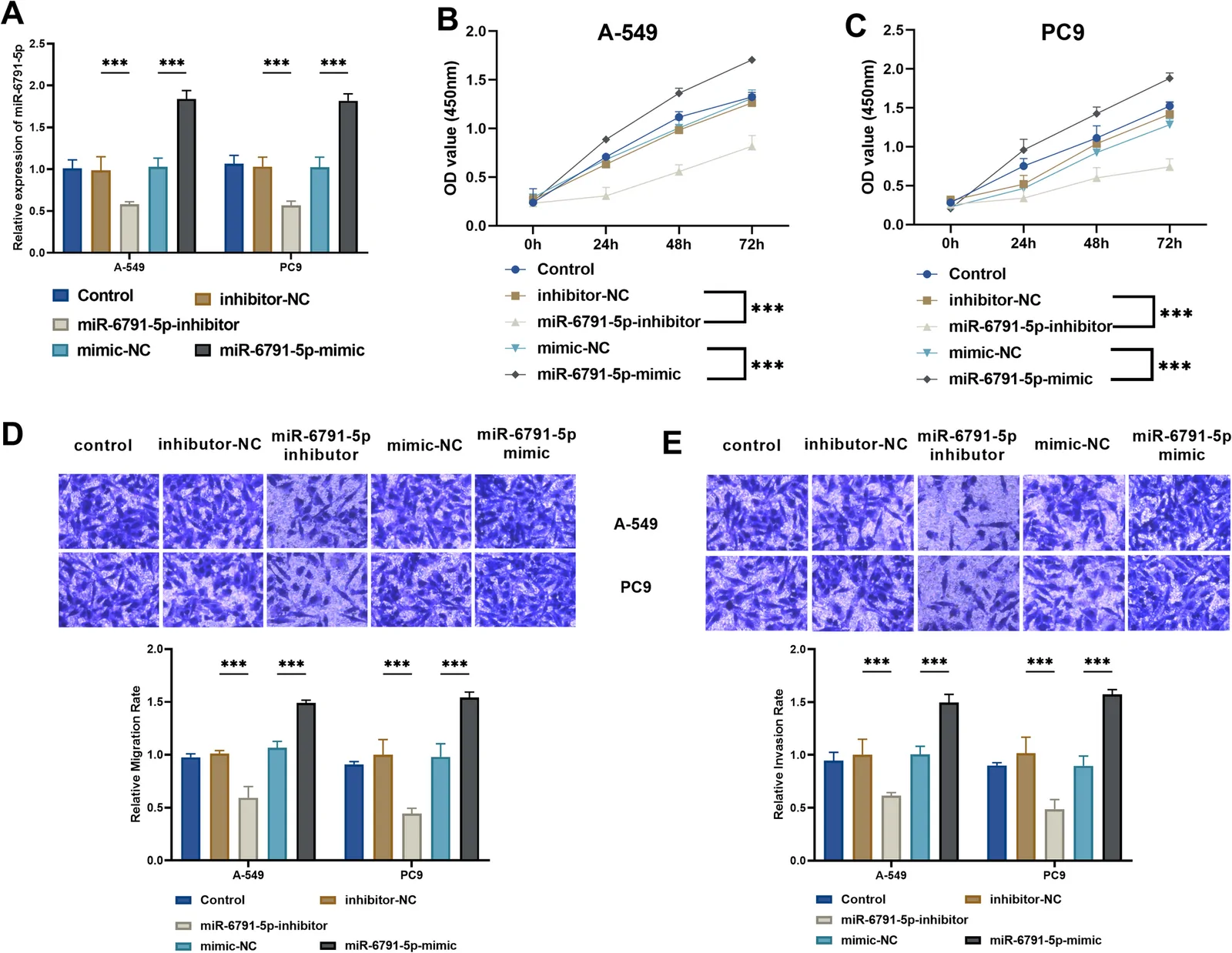
Functional effects of miR-6791-5p on LUAD cells. (**A**) Relative miR-6791-5p expression in A‑549 and PC9 cells following transfection with inhibitor or mimic. Cell proliferation assessed by CCK‑8 assay in A‑549 (**B**) and PC9 (**C**) cells. Migration (**D**) and invasion (**E**) capacities of A‑549 and PC9 cells after treatment. All quantitative data are presented as mean ± SD from three independent biological replicates (*n* = 3). Statistical significance was determined using one-way ANOVA (A, D, E) or two-way ANOVA (B, C).***p < 0.001

As evidenced by qPCR analysis in A‑549 and PC9 cells, transfection with the miR‑6791‑5p inhibitor resulted in a significant knockdown of its expression relative to both the Control and inhibitor‑NC groups (*p* < 0.001), while transfection with miR‑6791‑5p mimic markedly increased its expression relative to the Control and mimic‑NC groups (Fig. 2A, *p* < 0.001), confirming the successful validation of the transfection models.

Functional assays demonstrated that in both A-549 and PC9 cells, the OD_450_ values of the miR-6791-5p mimic group increased significantly over time and were consistently higher than Control group at all measured time points (Fig. 2B and C, *p* < 0.001). Conversely, cells transfected with the miR-6791-5p inhibitor exhibited significantly lower OD450 readings compared to the control group. Similarly, in migration and invasion assays, the miR-6791-5p mimic group exhibited significantly increased migration and invasion rates compared to the Control group, as clearly illustrated by the representative images of migrated and invaded cells (Fig. 2D and E, *p* < 0.001), whereas the miR-6791-5p inhibitor group showed significantly reduced migration and invasion capabilities. These results were consistent across both cell lines. Collectively, these in vitro functional assays demonstrate that miR-6791-5p promotes the proliferation, migration, and invasive capabilities of LUAD cells.

### miR‑6791‑5p directly targets and negatively regulates RASL12

**Fig. 3:**
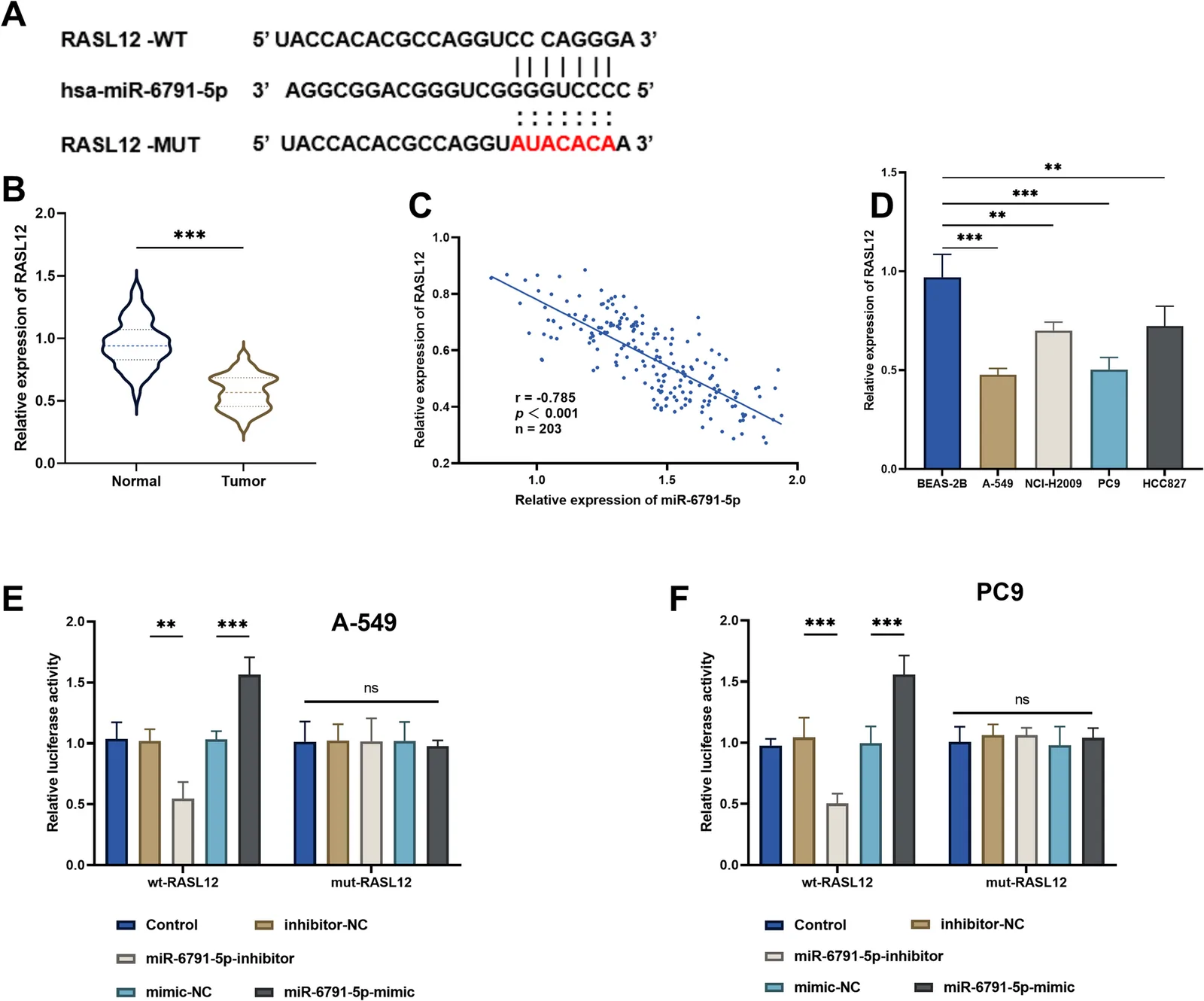
miR-6791-5p directly targets *RASL12* in LUAD. Predicted binding site of miR-6791-5p within the 3′-UTR of *RASL12* mRNA (**A**). *RASL12* expression levels in paired tumor and adjacent normal tissues (**B**). Negative correlation between miR-6791-5p and *RASL12* expression in clinical samples (**C**). *RASL12* expression across different cell lines (**D**). Dual-luciferase reporter assays confirming direct targeting by miR-6791-5p in A‑549 (**E**) and PC9 (**F**) cells. Data are presented as mean ± SD. Statistical significance was determined using paired Student’s t-test for (B) (*n* = 203), Pearson correlation for (C) (*n* = 203), and one-way ANOVA for (D, E, F) (*n* = 3 independent biological replicates). ns: not significant, **p < 0.01, ***p < 0.001

To explore the mechanism of miR‑6791‑5p in LUAD, bioinformatics analysis using TargetScan predicted a conserved binding site for miR-6791-5p within the 3′‑UTR of *RASL12* mRNA at positions 815–821 (Fig. 3A). As assessed by tissue‑level analysis of 203 paired samples, *RASL12* expression was markedly downregulated in LUAD tissues compared to their paired adjacent normal counterparts (Fig. 3B, *p* < 0.001).

Correlation analysis further revealed that miR‑6791‑5p expression in LUAD samples was significantly negatively correlated with *RASL12* expression (r = ‑0.785, *p* < 0.001, Fig. 3C). At the cellular level, *RASL12* expression in multiple LUAD cell lines was also significantly lower than in the normal bronchial epithelial cell line BEAS‑2B (Fig. 3D, *p* < 0.01). Dual‑luciferase reporter assay confirmed that transfection of miR‑6791‑5p mimic specifically inhibited the luciferase activity of the reporter vector carrying the wild‑type *RASL12* 3′‑UTR (WT‑*RASL12*), but had no significant effect on the vector with a binding‑site mutation (MUT‑*RASL12*), indicating that miR‑6791‑5p directly binds to *RASL12* (Fig. 3E and F).

To further confirm these findings at the translational level, we analyzed the CPTAC large-scale proteomic dataset via the UALCAN portal. Consistent with the mRNA transcript levels, the protein abundance of *RASL12* was significantly reduced in primary LUAD tissues (*n* = 111) compared to normal lung tissues (*n* = 111) (Supplementary Figure S1A). Furthermore, to address the limitation of our single-center clinical cohort, we evaluated the prognostic value of *RASL12* using the Kaplan-Meier Plotter database as an external validation cohort. The results demonstrated that low expression of *RASL12* was significantly associated with worse overall survival in a large, independent LUAD cohort (*p* < 0.001) (Supplementary Figure S1B). Therefore, these integrated results provide robust evidence that *RASL12* is a direct downstream target of miR-6791-5p and possesses significant prognostic value in LUAD.

### The independent tumor-suppressive role of RASL12 in LUAD cells

**Fig. 4:**
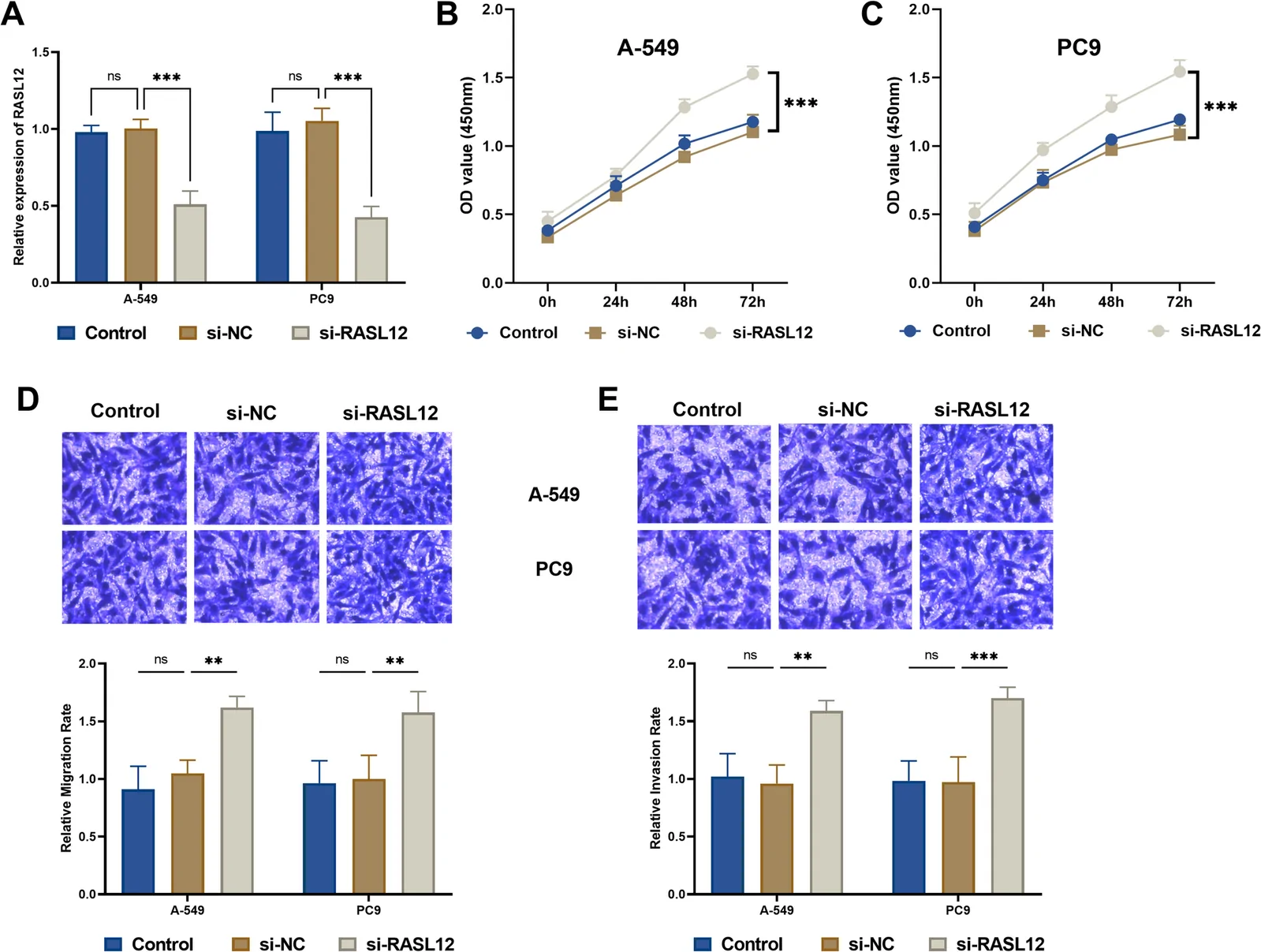
The independent tumor-suppressive role of *RASL12* in LUAD cells. *RASL12* expression in A‑549 and PC9 cells following transfection with si-*RASL12* alone (**A**). Cell proliferation assessed by CCK‑8 assay in A‑549 (**B**) and PC9 (**C**) cells. Relative migration (**D**) and invasion (**E**) capacities of A‑549 and PC9 cells. Data are presented as mean ± SD from three independent biological replicates (*n* = 3). Statistical significance was determined using one-way ANOVA (A, D, E) or two-way ANOVA (B, C). ns: not significant, **p < 0.01, ***p < 0.001

To determine the independent biological effect of *RASL12*, we performed loss-of-function assays using si-*RASL12* alone. As shown in Fig. 4A, transfection with si-*RASL12* effectively knocked down *RASL12* expression in both A-549 and PC9 cells (*p* < 0.001). Functional assays revealed that independent knockdown of *RASL12* significantly promoted cell proliferation (Fig. 4B and C, *p* < 0.001), migration (Fig. 4D, *p* < 0.01), and invasion (Fig. 4E, *p* < 0.01) compared to the control groups. These data suggest that *RASL12* plays a tumor-suppressive role in LUAD cells.

### RASL12 mediates the effect of miR‑6791‑5p on the malignant phenotype of LUAD cells

**Fig. 5:**
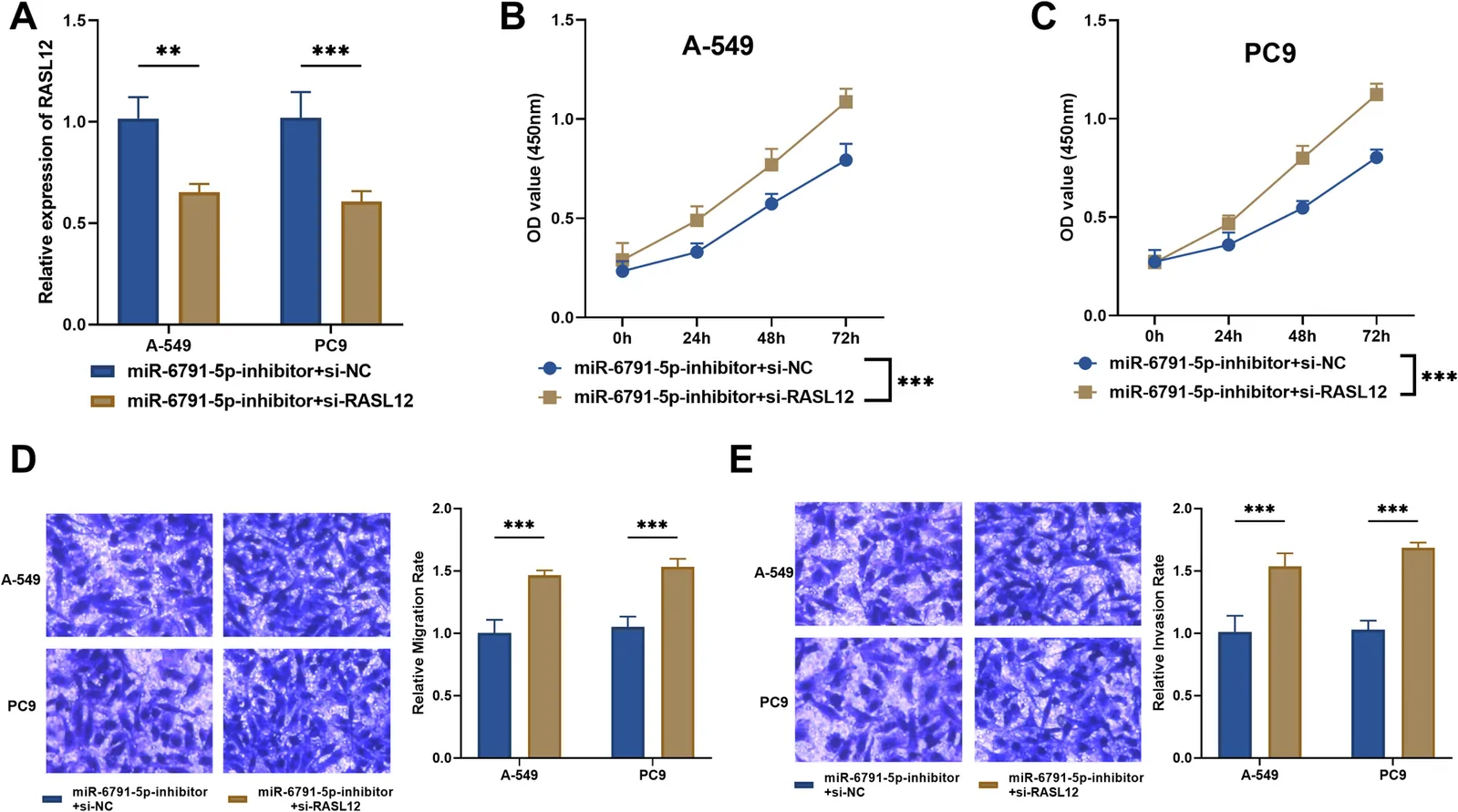
Rescue of the tumor‑promoting effects of miR‑6791‑5p by *RASL12* knockdown. *RASL12* expression in A‑549 and PC9 cells following co‑transfection with miR‑6791‑5p inhibitor and si‑*RASL12* (**A**). Cell proliferation assessed by CCK‑8 assay in A‑549 (**B**) and PC9 (**C**) cells after treatment. Relative migration (**D**) and invasion (**E**) capacities of A‑549 and PC9 cells. Data are presented as mean ± SD from three independent biological replicates (*n* = 3). Statistical significance was determined using one-way ANOVA (A, D, E) or two-way ANOVA (B, C). **p < 0.01, ***p < 0.001

To verify whether *RASL12* mediates the biological function of miR‑6791‑5p, we subsequently performed rescue experiments by knocking down *RASL12* in the context of miR-6791-5p inhibition. To confirm the knockdown efficiency in this co-transfection model, we assessed *RASL12* expression by qPCR from three independent experiments. As shown in Fig. 5A, transfection with si‑*RASL12* effectively knocked down *RASL12* expression, significantly reversing its upregulation that was initially induced by the miR‑6791‑5p inhibitor (*p* < 0.01).

Functional rescue experiments revealed that the malignant phenotype suppressed by miR-6791-5p inhibition could be partially restored through concurrent *RASL12* knockdown. Specifically, compared to the control (miR‑6791‑5p inhibitor + si‑NC), co‑knockdown (miR‑6791‑5p inhibitor + si‑*RASL12*) led to a significant recovery in cell proliferation (Fig. 5B and C), migration (Fig. 5D), and invasion (Fig. 5E). Therefore, these functional rescue experiments strongly suggest that *RASL12* partially mediates the oncogenic effects of miR-6791-5p on LUAD cell aggressiveness.

### RASL12 expression is associated with cell adhesion signaling pathways

**Fig. 6:**
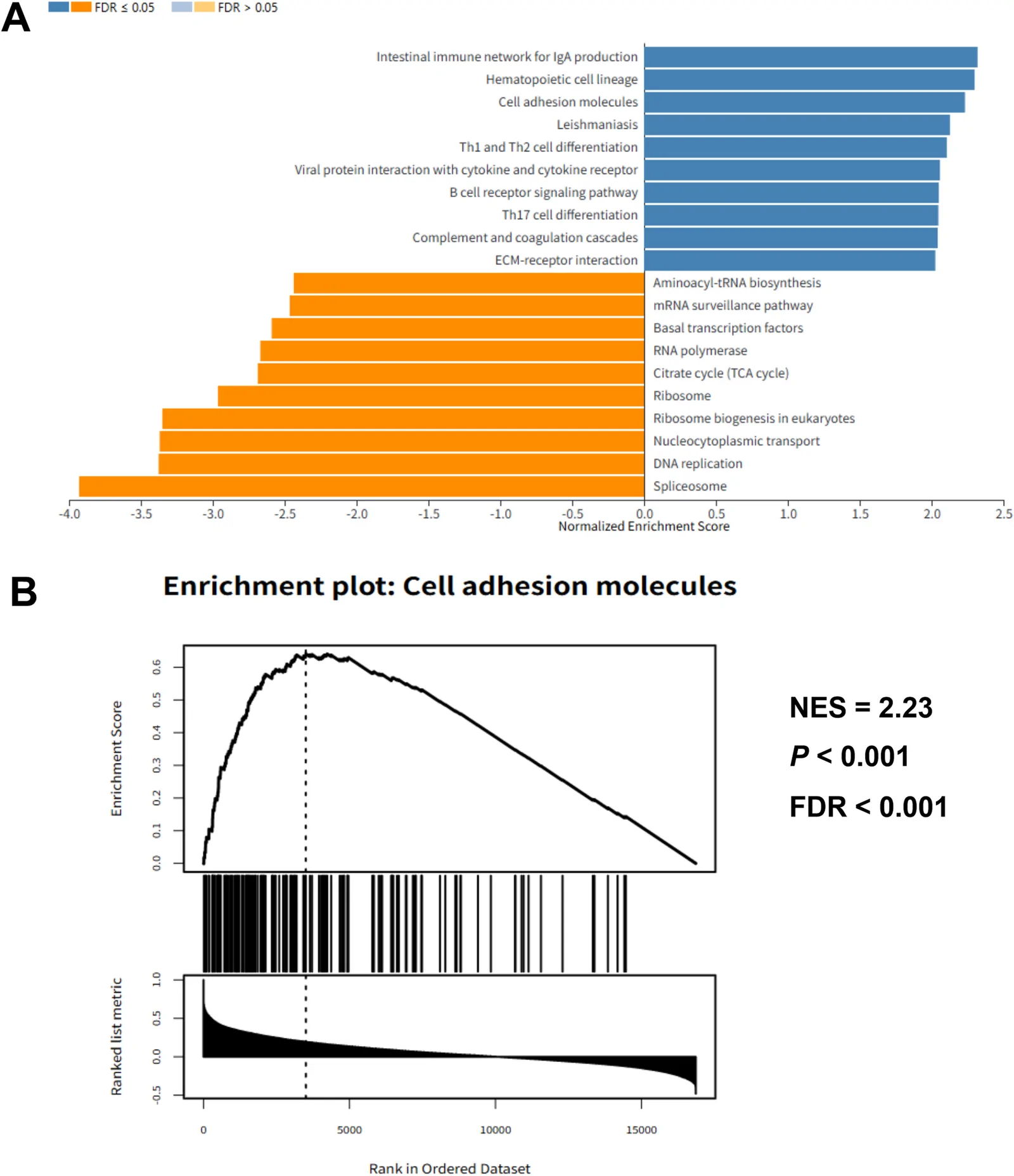
GSEA identifies cell adhesion molecules as a potential downstream mechanism of *RASL12*. Bar chart showing the top KEGG pathways significantly correlated with *RASL12* expression in the TCGA-LUAD cohort (*n* = 515) (**A**). Blue bars indicate positive enrichment, while orange bars indicate negative enrichment. GSEA enrichment plot for the “Cell adhesion molecules” pathway (**B**). The Normalized Enrichment Score (NES), nominal *p*-value, and FDR are indicated in the plot

To further investigate the molecular mechanism by which *RASL12* regulates LUAD progression, we performed an unbiased GSEA analysis using the LinkedOmics database based on the TCGA-LUAD cohort. The global enrichment profile (Fig. 6A) revealed that several pathways were significantly associated with *RASL12* expression. Notably, the “Cell adhesion molecules” pathway was among the most significantly enriched gene sets (Fig. 6B; NES = 2.23, *p* < 0.001, FDR < 0.001). Given our earlier observations that *RASL12* inhibition promotes cell migration and invasion, these bioinformatic findings suggest that the miR-6791-5p/*RASL12* axis may facilitate tumor metastasis primarily by modulating cell adhesion signaling dynamics.

## Discussion

The highly heterogeneous and aggressive biological behavior of LUAD is the core reason for the significant variation in prognosis and treatment response among patients [17–19]. Moving beyond traditional pathological staging to analyze the key mechanisms driving malignant progression from a molecular level has become an urgent clinical need. In this context, molecular markers such as miRNAs are emerging as promising avenues, given their high stability in bodily fluids and their ability to post-transcriptionally regulate gene expression broadly [20–22]. To situate our findings within the broader biomarker development landscape, the transition from basic discovery to reliable clinical application remains challenging [23]. Therefore, miR-6791-5p emerges as a potential supplementary biomarker that could provide predictive value beyond, rather than replacing, conventional clinical parameters.

Recent studies have highlighted the important regulatory roles of miRNAs in cancer. For example, miR‑6791‑5p has been associated with metastasis in cervical cancer [24], predicted within a ceRNA network in esophageal squamous cell carcinoma [25], and linked to hepatocyte apoptosis [26]. However, its direct clinical relevance in LUAD has remained unclear. In this study, we demonstrated that miR‑6791‑5p is significantly overexpressed in LUAD tissues, correlating with crucial clinicopathological parameters and serving as an independent predictor of poor survival. Functionally, it drives the proliferative, migratory, and invasive capacities of LUAD cells.

Based on the well-established mechanism that miRNAs typically function by targeting and regulating downstream mRNAs [27], this study further focused on identifying the specific targets of miR‑6791‑5p. A prior bioinformatics study by Li [14] predicted the involvement of the miR-6791-5p/*RASL12* axis in LUAD based on ceRNA network analysis. The present study provides functional validation and mechanistic characterization of this previously predicted interaction. By transitioning from in silico prediction to experimental validation, we confirmed that miR-6791-5p directly targets the *RASL12* 3’-UTR and promotes tumor progression by suppressing *RASL12* expression.

*RASL12*, a member of the RAS family, has been implicated as a potential tumor suppressor in a variety of cancers [15, 28, 29], and its expression positively correlates with tumor-suppressive networks [30]. Through bioinformatics prediction and dual-luciferase reporter assays, we confirmed that *RASL12* is a direct functional target of miR-6791-5p. Importantly, the molecular progression and therapeutic resistance of lung cancer are deeply intertwined with complex microenvironmental shifts, particularly metabolic reprogramming and immune evasion [31]. Recent multi-dimensional tumor biomarker characterizations across various malignancies further highlight that tumor progression relies on broad metabolic remodeling, such as altered lipid homeostasis or glycolysis, coupled with specific immune interactions like macrophage-oriented immune remodeling [32, 33]. In a similar context of complex signaling networks, targeting specific nodes like the MAPK or PI3K/AKT pathways crucially dictates tumor subtypes and patient risk profiles [34]. Just as other upstream regulators promote progression via the PI3K/AKT axis [35], our GSEA results revealed that *RASL12* downregulation is significantly associated with altered cell adhesion signaling dynamics. Therefore, the miR-6791-5p/*RASL12* axis likely coordinates with broader oncogenic signaling and microenvironmental networks to facilitate metastasis.

Despite these promising findings, we acknowledge several limitations in the present study. First, our functional assays were conducted exclusively in vitro. Future studies incorporating in vivo xenograft or orthotopic animal models are essential to confirm whether this axis drives tumor growth in a physiological context. Second, while we utilized the CPTAC dataset to confirm *RASL12* downregulation at the translational level, miRNA regulation often involves translational repression; thus, future independent studies utilizing Western blot or immunohistochemistry on expanded, multicenter clinical cohorts, particularly focusing on downstream phospho-protein analysis (e.g., p-ERK, p-AKT), will be necessary to strengthen our conclusions. Finally, our rescue experiments suggest that *RASL12* partially mediates the oncogenic effects of miR-6791-5p. Integrating multi-omics approaches in future research will help elucidate the full spectrum of downstream effectors. In summary, we identified miR-6791-5p as a novel prognostic indicator and an oncogenic factor in LUAD. By elucidating the specific mechanism through which it targets the tumor suppressor *RASL12*, we provide experimental evidence for a functional regulatory axis. Future investigations addressing the aforementioned limitations will be vital for translating these molecular insights into clinical applications.

## Conclusions

Collectively, our results identify miR‑6791‑5p as a potential prognostic marker and a promoter of tumor progression in LUAD. Mechanistically, our in vitro data suggest that it exerts its oncogenic effects at least partially by directly targeting and downregulating the tumor suppressor *RASL12*, thereby enhancing cancer cell proliferation, migration, and invasion. The miR-6791-5p/*RASL12* regulatory axis may thus represent a valuable mechanism for further investigation in LUAD pathobiology.

## Supplementary Information

Supplementary Material 1. Supplementary Figure S1. Downregulation of *RASL12* protein expression and its prognostic value. Relative protein abundance (Z-value) of *RASL12* in normal lung tissues (*n* = 111) and primary LUAD tissues (*n* = 111) based on the CPTAC dataset, analyzed via the UALCAN database (A). Kaplan-Meier survival curves demonstrating overall survival (OS) in a large independent LUAD cohort from the Kaplan-Meier Plotter database (B). Patients with low *RASL12* expression exhibited significantly poorer survival compared to those with high expression (*p* < 0.001, log-rank test).

Supplementary Material 2.

## Data Availability

The datasets used and/or analysed during the current study are available from the corresponding author on reasonable request.
