# Supplementary material for: Targeting RASL12 by miR-6791-5p fuels malignant progression in lung adenocarcinoma
Source: Hereditas. 2026 Jul 25;163:89. doi: 10.1186/s41065-026-00697-4 (PMC13491930; doi:10.1186/s41065-026-00697-4)
Supplement: Supplementary file 1 [file 41065_2026_697_MOESM2_ESM.docx]

Supplementary Table 1. Primer and oligonucleotide sequences used in this study

| Gene |  | Sequences (5´-3´) |
| --- | --- | --- |
| miR-6791-5p | Forward | GGTGCCCCCTGGGGCTGGGCAG |
|  | Reverse | CGACTCGATCCAGTCTCAGGGTCCGAGGTATTCGATCGAGTCGCACTTTTTTTTTTTTA |
| RASL12 | Forward | TCTTCAGCTGGTTGGGTCTTTAA |
|  | Reverse | CGACGGTCAGGATCATGTGT |
| U6 | Forward | CTCGCTTCGGCA GCACA |
|  | Reverse | AACGCTTCAC GAATTTGCGT |
| GAPDH | Forward | TCGGAGTCAACGGATTTGGT |
|  | Reverse | TTCCCGTTCTCAGCCTTGAC |
| miR-6791-5p mimics | Sense | CCCCUGGGGCUGGGCAGGCGGA |
|  | Antisense | CGCCUGCCCAGCCCCAGGGGNN |
| miR-6791-5p inhibitor |  | UCCGCCUGCCCAGCCCCAGG |
| si-RASL12 |  | Proprietary pool of 3 target-specific siRNAs (Santa Cruz, Cat. No. sc-90203) |
